# Supplementary material for: Leaky severe combined immunodeficiency in mice lacking non-homologous end joining factors XLF and MRI
Source: Aging (Albany NY). 2020 Dec 7;12(23):23578–97. doi: 10.18632/aging.202346 (PMC7762521; doi:10.18632/aging.202346)
Supplement: Supplementary Figure 1 [file aging-12-202346-s001.pdf]

SUPPLEMENTARY FIGURE

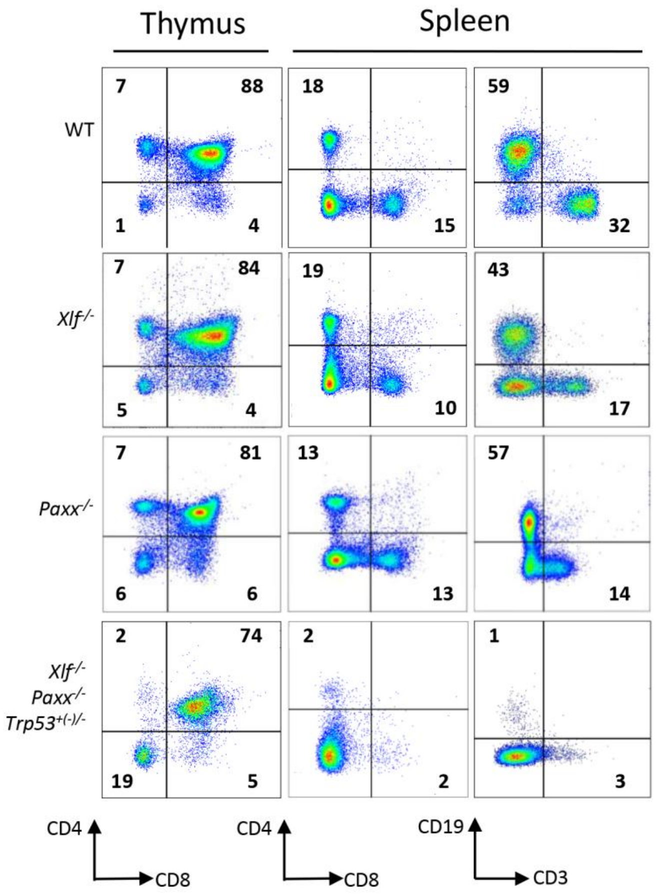

Supplementary Figure 1. B and T cell development in *Xlf$^{-/-}$Paxx$^{-/-}$Trp53$^{+(-)/-}$* mice. Examples of flow cytometric analysis of thymic and splenic T cell subsets and splenic CD19+ B cells. *Xlf$^{-/-}$Paxx$^{-/-}$Trp53$^{+(-)/-}$* is a combination of *Xlf$^{-/-}$Paxx$^{-/-}$Trp53$^{+/-}$* and *Xlf$^{-/-}$Paxx$^{-/-}$Trp53$^{-/-}$*.
