# Supplementary material for: Leaky severe combined immunodeficiency in mice lacking non-homologous end joining factors XLF and MRI
Source: Aging (Albany NY). 2020 Dec 7;12(23):23578–97. doi: 10.18632/aging.202346 (PMC7762521; doi:10.18632/aging.202346)
Supplement: Supplementary Tables [file aging-12-202346-s002.pdf]

SUPPLEMENTARY TABLES

Supplementary Table 1. Summary of splenic CD19+ B cells.

WT	*Xlf*$^{-/-}$	*Mri*$^{-/-}$	*Xlf*$^{-/-}$*Mri*$^{-/-}$*Trp53*$^{+/-}$	*Paxx*$^{-/-}$	*Xlf*$^{-/-}$*Paxx*$^{-/-}$*Trp53*$^{+(-)/-}$		*Paxx*$^{-/-}$*Mri*$^{-/-}$	*Dna-pkcs*$^{-/-}$
					Trp53$^{+/-}$	*Trp53*$^{-/-}$		
55.82	38.91	41.56	0.48	28.22	0.18	0.42	21.71	0.02
60.49	59.69	56.91	0.23	29.15	0.11	1.53	34.40	0.36
79.07	41.87	42.70	0.80	91.10	0.05	0.93	42.52	0.03
63.94	39.23	92.59	0.48	58.69	0.20		55.20	0.08
36.16	54.24	79.30	0.19	55.26	0.16		21.26	0.02
56.50	41.89	48.03		61.57	2.21		31.01	0.05
63.69	36.46	55.14		56.28	1.82		25.84	
42.85	37.87			39.59	0.58		15.51	
75.05	28.68			55.36	0.08		32.73	
67.60	39.08			61.29			58.43	
38.27	29.73			80.28			64.75	
79.47	47.11			61.29			36.96	
29.43	56.65			93.23			37.92	
52.58	44.10			61.86			63.63	
65.47	34.65						31.99	
62.80	30.79						22.31	
56.68	34.55						28.62	
57.03	50.48							
54.05	52.23							
75.79	36.41							

CD19+ splenocytes ($\times 10^6$) in WT, Xlf$^{-/-}$, Mri$^{-/-}$, Xlf$^{-/-}$Mri$^{-/-}$Trp53$^{+/-}$, Paxx$^{-/-}$, Xlf$^{-/-}$Paxx$^{-/-}$Trp53$^{+(-)/-}$ and Paxx$^{-/-}$Mri$^{-/-}$ mice. Dna-pkcs$^{-/-}$ mice were used as an immunodeficient control. Xlf$^{-/-}$Paxx$^{-/-}$Trp53$^{+(-)/-}$ is a combination of Xlf$^{-/-}$Paxx$^{-/-}$Trp53$^{+/-}$ and Xlf$^{-/-}$Paxx$^{-/-}$Trp53$^{-/-}$.

Supplementary Table 2. Summary of splenic CD3+ T cells.

WT	*Xlf*$^{-/-}$	*Mri*$^{-/-}$	*Xlf*$^{-/-}$*Mri*$^{-/-}$*Trp53*$^{+/-}$	*Paxx*$^{-/-}$	*Xlf*$^{-/-}$*Paxx*$^{-/-}$*Trp53*$^{+(-)/-}$		*Paxx*$^{-/-}$*Mri*$^{-/-}$	*Dna-pkcs*$^{-/-}$
					Trp53$^{+/-}$	*Trp53*$^{-/-}$		
41.61	15.06	23.01	5.55	34.34	0.13	0.41	26.93	0.38
37.93	36.01	36.61	3.31	21.89	0.52	1.42	24.53	0.23
42.64	33.33	23.12	3.05	38.84	0.42	0.84	36.66	0.03
29.44	39.67	32.84	2.36	23.51	0.26		41.18	0.09
15.25	30.11	30.74	1.55	31.11	0.92		15.22	0.02
22.62	15.19	19.45		16.26	0.25		18.83	0.07
20.41	49.15	25.38		14.86	0.71		20.21	
18.50	24.24			10.45	0.38		13.65	
27.19	17.04			14.62			17.85	
24.49	14.83			16.18			25.19	
13.86	15.41			21.20			28.55	
28.79	11.67			16.18			20.70	
19.05	15.90			24.62			16.90	
23.72	12.10			16.34			32.74	
22.75	19.17						17.44	
20.53	23.05						19.22	
27.01	17.94						18.25	
25.60	14.10							
35.90	12.52							
	14.06							
	20.54							
	21.25							
	42.82							
	14.81							

CD3+ splenocytes ($\times10^6$) in WT, Xlf$^{-/-}$, Mri$^{-/-}$, Xlf$^{-/-}$Mri$^{-/-}$Trp53$^{+/-}$, Paxx$^{-/-}$, Xlf$^{-/-}$Paxx$^{-/-}$Trp53$^{+(-)/-}$ and Paxx$^{-/-}$Mri$^{-/-}$ mice. Dna-pkcs$^{-/-}$ mice were used as an immunodeficient control. Xlf$^{-/-}$Paxx$^{-/-}$Trp53$^{+(-)/-}$ is a combination of Xlf$^{-/-}$Paxx$^{-/-}$Trp53$^{+/-}$ and Xlf$^{-/-}$Paxx$^{-/-}$Trp53$^{-/-}$.

Supplementary Table 3. Summary of splenic CD4+ T cells.

WT	*Xlf*$^{-/-}$	*Mri*$^{-/-}$	*Xlf*$^{-/-}$*Mri*$^{-/-}$*Trp53*$^{+/-}$	*Paxx*$^{-/-}$	*Xlf*$^{-/-}$*Paxx*$^{-/-}$*Trp53*$^{+(-)/-}$		*Paxx*$^{-/-}$*Mri*$^{-/-}$	*Dna-pkcs*$^{-/-}$
					Trp53$^{+/-}$	*Trp53*$^{-/-}$		
19.51	5.46	10.36	1.14	12.17	0.03	0.49	8.3	0.08
17.39	11.43	15.61	1.66	9.96	0.01	0.34	12.54	0.05
18.17	18.99	13.88	1.09	24.65	0.49	0.56	17.64	0.09
9.23	15.66	19.32	0.96	15.97	0.59		18.51	
13.63	14.9	17.57		19.38	0.35		6.66	
12.38	6.88	13.25			0.37		8.13	
11.89	21.81	14.42			0.37		8.92	
9.844					0.27		5.51	
8.961							9.67	
9.96							15.61	
15.85							18.01	
19.29							10.36	
16.42								
12.85								

CD4+ splenocytes (×10^6) in WT, Xlf$^{-/-}$, Mri$^{-/-}$, Xlf$^{-/-}$Mri$^{-/-}$Trp53$^{+/-}$, Paxx$^{-/-}$, Xlf$^{-/-}$Paxx$^{-/-}$Trp53$^{+(-)/-}$ and Paxx$^{-/-}$Mri$^{-/-}$ mice. Dna-pkcs$^{-/-}$ mice were used as an immunodeficient control. Xlf$^{-/-}$Paxx$^{-/-}$Trp53$^{+(-)/-}$ is a combination of Xlf$^{-/-}$Paxx$^{-/-}$Trp53$^{+/-}$ and Xlf$^{-/-}$Paxx$^{-/-}$Trp53$^{-/-}$.

Supplementary Table 4. Summary of splenic CD8+ T cells.

WT	*Xlf*$^{-/-}$	*Mri*$^{-/-}$	*Xlf*$^{-/-}$*Mri*$^{-/-}$*Trp53*$^{+/-}$	*Paxx*$^{-/-}$	*Xlf*$^{-/-}$*Paxx*$^{-/-}$*Trp53*$^{+(-)/-}$		*Paxx*$^{-/-}$*Mri*$^{-/-}$	*Dna-pkcs*$^{-/-}$
					Trp53$^{+/-}$	*Trp53*$^{-/-}$		
14.39	4.03	12.59	0.74	6.83	0.32	0.40	5.86	0.08
18.35	12.06	14.79	1.72	9.30	0.07	0.41	10.45	0.05
12.31	12.73	10.57	1.19	15.76	0.22	0.29	15.26	0.06
7.13	13.11	18.08	0.66	12.05	0.24		16.02	
9.70	9.67	15.62		14.11	0.02		5.60	
9.64	6.39	9.96			0.39		7.69	
7.96	14.92	12.09			0.20		7.16	
14.26							4.86	
12.96							7.28	
13.91							13.26	
11.62							14.87	
12.17							7.78	
10.88								
7.88								

CD8+ splenocytes (×10^6) in WT, Xlf$^{-/-}$, Mri$^{-/-}$, Xlf$^{-/-}$Mri$^{-/-}$Trp53$^{+/-}$, Paxx$^{-/-}$, Xlf$^{-/-}$Paxx$^{-/-}$Trp53$^{+(-)/-}$ and Paxx$^{-/-}$Mri$^{-/-}$ mice. Dna-pkcs$^{-/-}$ mice were used as an immunodeficient control. Xlf$^{-/-}$Paxx$^{-/-}$Trp53$^{+(-)/-}$ is a combination of Xlf$^{-/-}$Paxx$^{-/-}$Trp53$^{+/-}$ and Xlf$^{-/-}$Paxx$^{-/-}$Trp53$^{-/-}$.

Supplementary Table 5. Summary of thymic CD4+ T cells.

WT	*Xlf$^{-/-}$*	*Mri$^{-/-}$*	*Xlf$^{-/-}$Mri$^{-/-}$Trp53$^{+/-}$*	*Paxx$^{-/-}$*	*Xlf$^{-/-}$Paxx$^{-/-}$Trp53$^{+(-)/-}$*		*Paxx$^{-/-}$Mri$^{-/-}$*	*Dna-pkcs$^{-/-}$*
					Trp53$^{+/-}$	*Trp53$^{-/-}$*		
9.88	8.45	8.63	1.32	6.01	0.66	0.13	5.53	0.02
9.06	3.41	10.16	0.48	8.77	0.07	0.06	8.40	0.001
10.48	11.88	6.74	0.89	12.88	0.07	0.19	4.31	0.01
16.33	7.05	7.95	0.65	11.10	0.11		6.77	0.02
7.50	6.67	15.23	0.50	9.80	0.40		6.85	
10.64	11.66	17.57			0.29		13.08	
7.12		15.94			0.19		9.52	
4.33		10.99			0.13		6.86	
2.55		15.88					12.67	
11.74		9.21					12.97	
13.15								
14.54								
14.41								
12.07								
11.39								
9.70								

CD4+ thymocytes (×10^6) in WT, Xlf$^{-/-}$, Mri$^{-/-}$, Xlf$^{-/-}$Mri$^{-/-}$Trp53$^{+/-}$, Paxx$^{-/-}$, Xlf$^{-/-}$Paxx$^{-/-}$Trp53$^{+(-)/-}$ and Paxx$^{-/-}$Mri$^{-/-}$ mice. Dna-pkcs$^{-/-}$ mice were used as an immunodeficient control. Xlf$^{-/-}$Paxx$^{-/-}$Trp53$^{+(-)/-}$ is a combination of Xlf$^{-/-}$Paxx$^{-/-}$Trp53$^{+/-}$ and Xlf$^{-/-}$Paxx$^{-/-}$Trp53$^{-/-}$.

Supplementary Table 6. Summary of thymic CD8+ T cells.

WT	*Xlf$^{-/-}$*	*Mri$^{-/-}$*	*Xlf$^{-/-}$Mri$^{-/-}$Trp53$^{+/-}$*	*Paxx$^{-/-}$*	*Xlf$^{-/-}$Paxx$^{-/-}$Trp53$^{+(-)/-}$*		*Paxx$^{-/-}$Mri$^{-/-}$*	*Dna-pkcs$^{-/-}$*
					Trp53$^{+/-}$	*Trp53$^{-/-}$*		
2.70	2.39	3.48	0.64	4.34	0.18	0.19	1.08	0.02
2.00	1.83	3.43	0.33	2.93	0.06	0.09	1.85	0.001
3.84	3.40	1.82	0.91	5.51	0.28	0.21	1.33	0.01
4.69	2.60	2.72	0.29	4.52	0.51		2.24	0.01
1.47	2.60	7.14	0.67	4.7	0.15		3.07	
2.11	3.59	6.40			0.23		6.2	
5.07		5.45			0.60		4.03	
3.26		4.28			0.49		3.16	
1.98							5.14	
3.91							5.11	
13.59								
11.68								
13.78								
3.47								
5.36								
4.90								
2.25								
4.56								

CD8+ thymocytes (×10^6) in WT, Xlf$^{-/-}$, Mri$^{-/-}$, Xlf$^{-/-}$Mri$^{-/-}$Trp53$^{+/-}$, Paxx$^{-/-}$, Xlf$^{-/-}$Paxx$^{-/-}$Trp53$^{+(-)/-}$ and Paxx$^{-/-}$Mri$^{-/-}$ mice. Dna-pkcs$^{-/-}$ mice were used as an immunodeficient control. Xlf$^{-/-}$Paxx$^{-/-}$Trp53$^{+(-)/-}$ is a combination of Xlf$^{-/-}$Paxx$^{-/-}$Trp53$^{+/-}$ and Xlf$^{-/-}$Paxx$^{-/-}$Trp53$^{-/-}$.

Supplementary Table 7. Summary of thymic CD4+CD8+ double positive T cells.

WT	*Xlf*$^{-/-}$	*Mri*$^{-/-}$	*Xlf*$^{-/-}$*Mri*$^{-/-}$*Trp53*$^{+/-}$	*Paxx*$^{-/-}$	*Xlf*$^{-/-}$*Paxx*$^{-/-}$*Trp53*$^{+(-)/-}$		*Paxx*$^{-/-}$*Mri*$^{-/-}$	*Dna-pkcs*$^{-/-}$
					Trp53$^{+/-}$	*Trp53*$^{-/-}$		
154.05	48.40	160.60	20.69	132.56	2.68	6.22	75.37	0.17
141.52	73.77	133.62	11.40	161.22	7.21	6.54	184.27	0.002
230.74	163.07	14.22	21.18	151.30	6.79	5.14	122.11	0.0002
147.74	95.47	165.78	17.78	208.39	3.39		105.44	0.001
138.62	115.71	154.74	17.71	202.99	3.72		88.37	0.002
98.78	115.77	193.15		161.06	4.43		168.00	
115.10	174.36	102.72		171.03	11.90		122.32	
66.71	144.88			100.87	5.18		87.20	
102.13	160.88			175.18			136.82	
162.51	105.29			197.59			153.47	
126.04	155.83						114.36	
79.43	90.46						136.39	
140.22	118.17						55.65	
146.71	183.46						96.22	
119.15	158.35							
	172.50							
	146.98							
	101.50							
	143.61							
	114.38							
	132.49							
	105.96							
	136.45							
	162.14							

CD4+CD8+ thymocytes (×10^6) in WT, Xlf$^{-/-}$, Mri$^{-/-}$, Xlf$^{-/-}$Mri$^{-/-}$Trp53$^{+/-}$, Paxx$^{-/-}$, Xlf$^{-/-}$Paxx$^{-/-}$Trp53$^{+(-)/-}$ and Paxx$^{-/-}$Mri$^{-/-}$ mice. Dna-pkcs$^{-/-}$ mice were used as an immunodeficient control. Xlf$^{-/-}$Paxx$^{-/-}$Trp53$^{+(-)/-}$ is a combination of Xlf$^{-/-}$Paxx$^{-/-}$Trp53$^{+/-}$ and Xlf$^{-/-}$Paxx$^{-/-}$Trp53$^{-/-}$.

Supplementary Table 8. Summary of IgM+ B cells in bone marrow.

WT	*Xlf*$^{-/-}$	*Mri*$^{-/-}$	*Xlf*$^{-/-}$*Mri*$^{-/-}$*Trp53*$^{+/-}$	*Paxx*$^{-/-}$	*Xlf*$^{-/-}$*Paxx*$^{-/-}$*Trp53*$^{+(-)/-}$		*Paxx*$^{-/-}$*Mri*$^{-/-}$
					Trp53$^{+/-}$	*Trp53*$^{-/-}$	
19.80	7.82	17.10	3.04	17.8	2.19	3.26	15.90
16.70	11.00	16.30	1.47	14.3	2.53	4.01	16.70
18.60	8.47	16.00	1.42	15.9	4.09	3.55	14.30
10.10	6.04	15.80	1.10	11.9	0.37		14.00
14.10	7.68	13.80	1.57		4.60		10.10
11.60	6.06	11.50			4.35		9.82
12.40	12.40	14.90			1.50		7.34
13.90	10.60	13.00			3.76		8.22
14.10	5.79				5.19		14.60
14.50					6.20		14.10
13.70							
10.10							
12.10							

Frequencies (%) of B220+CD43-IgM+ B cells in WT, Xlf$^{-/-}$, Mri$^{-/-}$, Xlf$^{-/-}$Mri$^{-/-}$Trp53$^{+/-}$, Paxx$^{-/-}$, Xlf$^{-/-}$Paxx$^{-/-}$Trp53$^{+(-)/-}$ and Paxx$^{-/-}$Mri$^{-/-}$ mice. Xlf$^{-/-}$Paxx$^{-/-}$Trp53$^{+(-)/-}$ is a combination of Xlf$^{-/-}$Paxx$^{-/-}$Trp53$^{+/-}$ and Xlf$^{-/-}$Paxx$^{-/-}$Trp53$^{-/-}$.

Supplementary Table 9. Summary of progenitor B cells in bone marrow.

WT	*Xlf*$^{-/-}$	*Mri*$^{-/-}$	*Xlf*$^{-/-}$*Mri*$^{-/-}$*Trp53*$^{+/-}$	*Paxx*$^{-/-}$	*Xlf*$^{-/-}$*Paxx*$^{-/-}$*Trp53*$^{+(-)/-}$		*Paxx*$^{-/-}$*Mri*$^{-/-}$
					Trp53$^{+/-}$	*Trp53*$^{-/-}$	
6.00	21.6	3.74	24.90	8.92	33.70	20.10	7.53
7.47	8.73	4.31	14.50	4.40	28.40	24.00	7.04
3.38	17.5	9.53	17.20	4.07	23.20	25.60	8.64
6.96	10.9	7.03	14.90	3.81	21.90		10.9
5.02	9.61	6.73	19.00		26.80		9.00
5.49	7.99	6.58			27.10		6.28
2.75	5.31	4.17			25.10		6.76
4.25	7.16	4.28			17.30		6.20
7.58	6.08				22.20		3.32
7.47					25.50		4.06
5.03							
7.72							
8.29							

Frequencies (%) of B220+CD43+IgM- pro-B cells in WT, Xlf$^{-/-}$, Mri$^{-/-}$, Xlf$^{-/-}$Mri$^{-/-}$Trp53$^{+/-}$, Paxx$^{-/-}$, Xlf$^{-/-}$Paxx$^{-/-}$Trp53$^{+(-)/-}$ and Paxx$^{-/-}$Mri$^{-/-}$ mice. Xlf$^{-/-}$Paxx$^{-/-}$Trp53$^{+(-)/-}$ is a combination of Xlf$^{-/-}$Paxx$^{-/-}$Trp53$^{+/-}$ and Xlf$^{-/-}$Paxx$^{-/-}$Trp53$^{-/-}$.

Supplementary Table 10. Lymphocytic development in the *Xlf*$^{-/-}$*Mri*$^{-/-}$*Trp53*$^{+/+}$ mouse.

Splenocytes (×10^{6})				Thymocytes (×10^{6})			Cell populations (%) in bone marrow	
CD19+ B cells	CD3+ T cells	CD4+ T cells	CD8+ T cells	CD4+ T cells	CD8+ T cells	CD4+CD8+ T cells	IgM+ B cells	Pro-B cells
0.11	0.80	0.53	0.41	0.42	0.41	15.70	2.71	19.40

Summary of splenic (×10^{6}) B- and T cells; and T cell subpopulations in the thymus (×10^{6}). Frequencies (%) in bone marrow of B220+CD43-IgM+ B cells and B220+CD43+IgM- pro-B cells.
